# Non-specific diagnoses are frequent in patients hospitalized after calling 112 and their mortality is high – a register-based Danish cohort study

**DOI:** 10.1186/s13049-020-00768-z

**Published:** 2020-07-22

**Authors:** Frederikke Vestergaard Nielsen, Mette Rønn Nielsen, Jesper Amstrup, Ida Lund Lorenzen, Torben A. Kløjgaard, Emil Færk, Henrik Bøggild, Erika Frischknecht Christensen

**Affiliations:** 1 Centre for Prehospital and Emergency Research, Department of Clinical Medicine, Aalborg University, Søndre Skovvej 15, 9000 Aalborg, Denmark; 2 Public Health and Epidemiology Group, Department of Health Science and Technology, Aalborg University, Niels Jernes Vej 14, 9220 Aalborg, Denmark; 3 Unit of Clinical Biostatistics, Aalborg University Hospital, Søndre Skovvej 15, 9000 Aalborg, Denmark; 4 Clinic for Internal and Emergency Medicine, Aalborg University Hospital, Hobrovej 18-22, 9000 Aalborg, Denmark; 5 Emergency Medical Services, North Denmark Region, Hjulmagervej 20, 9000 Aalborg, Denmark

**Keywords:** Ambulances, Emergency medical services, Non-specific diagnoses, Mortality, Outcome

## Abstract

**Background:**

The number of patients calling for an ambulance increases. A considerable number of patients receive a non-specific diagnosis at discharge from the hospital, and this could imply less serious acute conditions, but the mortality has only scarcely been studied. The aim of this study was to examine the most frequent sub-diagnoses among patients with hospital non-specific diagnoses after calling 112 and their subsequent mortality.

**Methods:**

A historical cohort study of patients brought to the hospital by ambulance after calling 112 in 2007–2014 and diagnosed with a non-specific diagnosis, chapter R or Z, in the International Classification of Diseases, 10th edition (ICD-10). 1-day and 30-day mortality was analyzed by survival analyses and compared by the log-rank test.

**Results:**

We included 74,847 ambulance runs in 53,937 unique individuals. The most frequent diagnoses were ‘unspecified disease’ (Z039), constituting 47.0% (n 35,279). In children 0–9 years old, ‘febrile convulsions’ was the most frequent non-specific diagnosis used in 54.3% (n 1602). Overall, 1- and 30-day mortality was 2.2% (n 1205) and 6.0% (n 3258). The highest mortality was in the diagnostic group ‘suspected cardiovascular disease’ (Z035) and ‘unspecified disease’ (Z039) with 1-day mortality 2.6% (n 43) and 2.4% (n 589), and 30 day mortality of 6.32% (n 104) and 8.1% (n 1975).

**Conclusion:**

Among patients calling an ambulance and discharged with non-specific diagnoses the 1- and 30-day mortality, despite modest mortality percentages lead to a high number of deaths.

## Background

In Denmark and other western countries, the number of emergency patients increases, raising a discussion of avoidable ambulance use and emergency department visits in an editorial of BMJ: *“Almost 1.5m hospital admissions could have been avoided last year”*, suggesting that *“Ambulances should treat more and transport fewer …*” [1, 2]. However, actual knowledge about the disease pattern in patients attended by emergency ambulances is scarce, due to limited prehospital patient data. In the North Denmark Region, we established a linkage between patient data from ambulance runs and hospital diagnoses [3], examining patients brought to the hospital by ambulance after calling the national emergency number 112. The two non-specific diagnostic groups, R (‘symptoms, signs and abnormal clinical and laboratory findings, not elsewhere classified’) and Z (‘factors influencing health status and contact with health services’) in the International Classification of Diseases, 10th edition (ICD-10) [4], together constituted one-third of the hospital diagnoses given to the included patients [3]. It may be tempting to regard emergency ambulances to patients with non-specific diagnoses as unnecessary ambulance runs. The 30-day mortality in the above-mentioned study among patients with R and Z diagnoses was 4.3 and 1.3%, which not seems high compared to 14.7% in circulatory diseases and 11.6% in respiratory diseases. However, due to the large number of patients assigned a non-specific diagnosis, the total number of deaths corresponded to 20% (1377 of total 6901 deaths) of all deaths in the entire 112-patient population. Thus, in this paper, we aim to examine the diagnostic pattern and mortality for the most frequent sub-diagnoses within the R and Z chapters.

## Methods

### Study design

This study is a historical cohort study in the North Denmark Region of patients brought to the hospital by ambulance after calling the national emergency number 112 and diagnosed with a non-specific diagnosis with the aim to investigate 1 and 30-days mortality. We define non-specific diagnoses as the two main chapters R and Z of ICD-10. We linked data for patients from the medical records in the ambulances with the hospital diagnoses during the eight-year period, January 12,007 – December 312,014 and followed them for death within the next month. The study is reported according to STROBE guidelines.

### Study setting

North Denmark Region encompasses 10% of the Danish population (589,000 inhabitants) [5]. In each of the five health care regions in Denmark, a public prehospital organization runs the Emergency Medical Coordination Center (EMCC) assessing calls to the national emergency number, 112, concerning illness and injury, and assess the level of urgency [6]. In 2006 the North Denmark Region was the first to implement an electronic prehospital medical record (amPHI®). The patient data are accessible for the healthcare staff at the EMCC and incorporated into the hospitals’ electronic medical record [3].

### Participants

We included 74,847 patients brought to the hospital by ambulance after calling 112 in 2007–2014 in the North Denmark Region and diagnosed at the hospital with a non-specific ICD-10 diagnosis. Each ambulance dispatch followed by hospital discharge represented one patient contact, so each patient might be included more than once in the description of the non-specific diagnoses. The cohort has previously been described [3]. Due to the linkage, we had no loss-to-follow-up for the participants.

### Outcomes, data sources and variables

Outcome was measured as 1- and 30-day mortality and in these analyses, we only included unique individuals (53,937) based on the most recent hospital contact.

We retrieved prehospital data from the ambulance dispatch system and the prehospital medical record. Patients with hospital contact receive an ICD-10 diagnosis at discharge [7]. The regional Patient Administration System (PAS) links diagnoses from the hospitals, EMCC ambulance dispatch data, patient data from the prehospital medical record and hospital data using a unique 10-digit civil personal registration (CPR) number. If less than 5 h was recorded between the registration in the EMCC ambulance dispatch data and the time of hospital arrival in PAS, we defined it to represent the hospital contact connected to the included ambulance run. Similarly, we defined it as the same hospital stay if less than 2 h was recorded between the first and the next department contact. Sex, date of birth, and eventual date of death were extracted from the Danish Civil Registration System [8]. This information is generally regarded as complete and valid [8]. Age was calculated at the date of ambulance run. Age is defined as years from the date of birth and divided into four age groups (0–9, 10–29, 30–59 and 60 years and older). Since death is only registered by the date and not by the hour, we defined 1-day mortality as death on the same day or the day after calling 112, and 30-day mortality as death up until 30 days after admission.

We shortened the naming of ICD-10 diagnoses of non-specific diagnoses (Table 1).

**Table 1:** List of abbreviations of ICD-10 diagnoses

ICD-10 diagnosis	Abbreviation
Other and unspecified convulsions (R568)	Unspecified convulsions (R568)
Other ill-defined and unspecified causes of mortality (R99)	Causes of mortality, UNS (R99)
Observation for suspected nervous system disorder (Z033)	Suspected nervous disease (Z033)
Observation for other suspected cardiovascular diseases (Z035)	Suspected cardiovascular disease (Z035)
Observation for other suspected diseases and conditions (Z038)	Observation for disease (Z038)
Observation for suspected disease or condition, unspecified (Z039)	Unspecified disease (Z039)
Examination and observation following transport accident (Z041)	Observation following transport accident (Z041)

The ICD-10 sub-diagnoses describing death such as ‘causes of mortality, UNS’ (R99), ‘respiratory arrest’ (R092), ‘other sudden death, cause unknown’ (R96) and ‘unattended death’ (R98) were included.

### Statistical methods

We described the distribution of sub-diagnoses by numbers and proportions. We analyzed mortality by survival analyses with censoring at death or after 30 days, and mortality was reported as proportion and the cumulative numbers of deaths. Comparison of survival curves was made by the log-rank test.

In the mortality analysis, a 95% confidence interval (CI) was estimated for proportions. A sensitivity analysis was performed to examine if the inclusion of the ICD-10 diagnoses defined as being dead (= ‘unspecified causes of death’ (R99) ‘respiratory arrest’ (R092), ‘other sudden death, cause unknown’ (R96) and ‘unattended death’ (R98)) made a difference in 30-day mortality.

Statistical analyses were performed using Stata V.15.1 (STATA Corporation, College Station, USA).

## Results

**Fig. 1:**
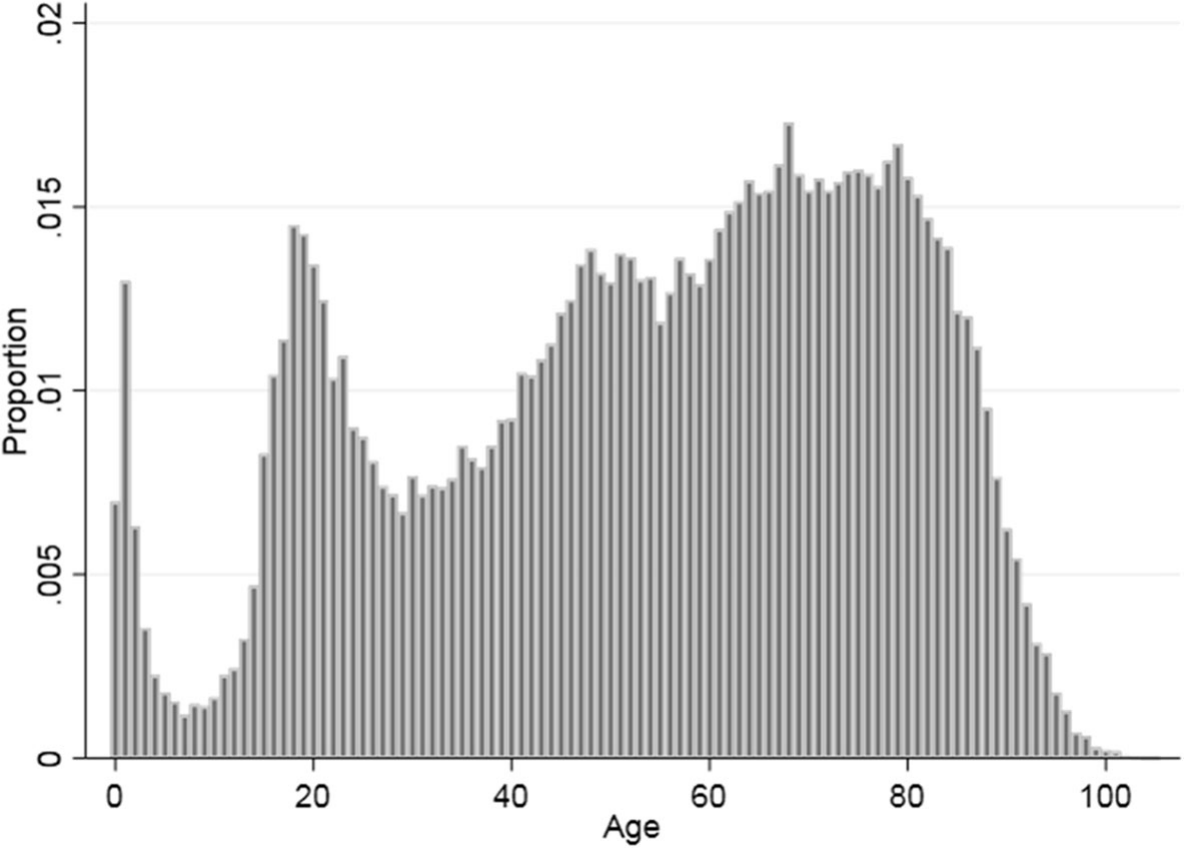
Age distribution among 74,847 patients with a non-specific hospital diagnosis after calling 112 in 2007–2014 in the North Denmark Region

Seventy-four thousand eight hundred forty-seven patients, including 53,973 (72.1%) unique individuals, were brought to the hospital by ambulance after calling 112 and diagnosed with a non-specific diagnosis at discharge. Men constituted 53.9% and the median age was 57 years (interquartile range 38). Figure 1 shows the age distribution.

The sub-diagnosis ‘obs suspected diseases’ (Z03) constituted 64% of all the non-specific diagnoses and at the next level, the most frequent sub-diagnosis was ‘unspecified disease’ (Z039) given to 47% (n 35,279).

‘Unspecified disease’ (Z039) was the most frequent diagnosis in all age groups except in 0–9 years, where 54.3% of patients had the diagnosis ‘febrile convulsions’ (R560) (Table 2).

**Table 2:** Age distribution for the five most frequent sub-diagnoses according to ICD-10 among each age group for patients given a non-specific hospital diagnosis after calling 112 in 2007–2014 in the North Denmark Region

Subdiagnoses	Age groups									
	0–9 years		10–29 years		30–59 years		60+ years		Total	
	N	Percent	N	Percent	N	Percent	N	Percent	N	Percent
Unspecified disease (Z039)	311	10.5%	5102	40.7%	11,401	46.5%	18,465	52.9%	35,279	47%
Observation for disease (Z038)	325	11.0%	959	7.7%	1762	7.2%	2088	6.0%	5134	6.9%
Syncope and collapse (R55)	–	–	982	7.8%	1257	5.1%	2478	7.1%	4767	6.4%
Suspected nervous disease (Z033)	180	6.1%	–	–	1030	4.2%	1146	3.3%	2969	4.0%
Suspected cardiovascular disease (Z035)	–	–	–	–	947	3.9%	1493	4.3%	2623	3.5%
Observation following transport accident (Z041)	–	–	788	6.3%	–	–	–	–	1742	2.3%
Febrile convulsions (R560)	1602	54.3%	–	–	–	–	–	–	1608	2.1%
Unspecified convulsions (R568)	104	3.5%	–	–	–	–	–	–	806	1.1%
Others	428	14.5%	4396	35.1%	8104	33.1%	9207	26.4%	19,919	26.6%
Total	2948	100.0%	12,524	100.0%	24,500	100.0%	34,875	100.0%	74,847	100.0%

The overall 1-day and 30-day mortality was 2.2% (95% CI 2.1–2.4) and 6.0% (95% CI 5.8–6.2) (Table 3). The highest 1-day mortality was seen in ‘suspected cardiovascular disease’ (Z035) with 2.6% (95% CI 2.2–2.6) and ‘unspecified disease’ (Z039) with 2.4% (95%CI 1.9–3.5). The highest 30-day mortality was found in the group ‘unspecified disease’ (Z039), with 8.1% (95%CI 7.7–8.4), while ‘suspected cardiovascular disease’ (Z035) had a mortality of 6.3% (95%CI 5.2–7.6). The largest number of deaths were seen among ‘unspecified disease’ (Z039) constituting 49% (589/1205) of all deaths at day one and 61% (1975/3258) at day 30.

**Table 3:** Days 1 and 30 mortality for the five most frequent sub-diagnoses according to ICD-10 among patients given a non-specific hospital diagnosis after calling 112 in 2007–2014 in the North Denmark Region

Subdiagnoses	1-day mortality		30-day mortality	
	N	Percent (95% CI)	N	Percent (95% CI)
Unspecified disease (Z039)	589	2.41 (2.22 to 2.61)	1975	8.08 (7.74 to 8.43)
Suspected cardiovascular disease (Z035)	43	2.61 (1.90 to 3.50)	104	6.32 (5.19 to 7.60)
Cramp and spasm (R252)	2	0.98 (0.12 to 3.48)	9	4.39 (2.03 to 8.18)
Suspected nervous disease (Z033)	8	0.95 (0.41 to 1.85)	36	4.26 (3.00 to 5.84)
Observation for disease (Z038)	33	0.89 (0.61 to 1.25)	139	3.75 (3.16 to 4.41)
Others	530	2.30 (2.11 to 2.50)	995	4.32 (4.05 to 4.58)
Total (all sub-diagnoses)	1205	2.23 (2.11 to 2.36)	3258	6.04 (5.84 to 6.24)

The mortality of the five most frequent non-specific sub-diagnoses was compared and they differed statistically (log-rank test, *p* < 0.00005).

The ICD-10 sub-diagnoses describing death such as ‘unspecified causes of death’ (R99), ‘respiratory arrest’ (R092), ‘other sudden death, cause unknown’ (R96) and ‘unattended death’ (R98), constituted less than 1% of the 74,847 patients. ‘Causes of mortality, UNS’ (R99) and ‘respiratory arrest’ (R092) constituted 320 patients and 37 patients respectively. The diagnoses ‘other sudden death, cause unknown’ (R96) and ‘unattended death’ (R98) were given to less than three patients. A sensitivity analysis showed that the inclusion of these diagnoses did not change mortality (data not shown).

## Discussion

In this population-based study of nearly 80,000 acute ambulance patients, assigned non-specific R or Z diagnoses at the hospital, the vast majority, two-third had non-specific sub-diagnosis such as ‘obs suspected diseases’ (Z03). Among these, nearly half of all patients were labeled ‘unspecified disease’ (Z039). Mortality was highest for patients diagnosed as ‘unspecified disease’ (Z039), where 8% died within 30 days, exceeding even the mortality among ‘suspected cardiovascular disease’ (Z035). Though some patients with ‘causes of mortality, UNS’ (R99) could have died from cardiovascular disease, this is a high figure. Moreover, the number of deaths due to the nonspecific sub-diagnoses constituted a very large part of the total number of deaths.

The population-based design and large study population are strengths of this study. Each patient might be included more than once since we examined the total number of patient contacts. The Danish population has free and equal access to prehospital care, minimizing the risk of financial resources influencing the inclusion of patients. The linkage of prehospital- and hospital data and the complete follow up is another strength, minimizing the risk of selection and information bias.

A weakness is that only patients with known CPR number were included to allow linking prehospital and hospital data sources. We were not able to determine the number of patients excluded due to this, and missing CPR numbers are a well-known weakness in prehospital emergency service studies. This may introduce bias in either direction, since patients without known CPR number may be more or less ill [7].

Some patients may have moved out of the region during the study, which may result in incomplete mortality reporting. However, with the large size of the cohort and the relatively short period, we find this to be of minor influence.

Patients hospitalized more than once might have received different non-specific diagnoses each time. Mortality was analyzed from the most recent hospital contact, since the patients are ‘immortal’ in the time between two hospital contacts, hereby excluding the first non-specific diagnosis.

The sub-diagnoses describing death constituted 11% of the 30-day mortality. Overall, the diagnoses constituted less than 1% of the total number of diagnoses. Since the sensitivity analysis showed no considerable differences in mortality, these diagnoses are assumed to have no influence on the results.

Our study only included patients transported to the hospital by ambulance, and are not directly generalizable to all prehospital patients.

Several studies found that non-specific diagnoses represent a considerable part among acute patients: among patients taken to hospital by ambulance after calling 112 [3], among acute medical admissions [9, 10] and among all patients with contact to emergency departments [11]. Recently also, a German study showed that R-diagnoses (ICD-10 Chapter 18) was the third frequent diagnosis constituting 11–12% among patients admitted to hospital with ambulance [12]. Another Danish study found that ‘unspecified disease’ (Z039) and ‘observation for disease’ (Z038) comprised 76% of all Z03-codes [13]. These findings agree with our study finding that two-thirds of all non-specific diagnoses consisted of Z03-codes, with the most frequent diagnoses unspecified diseases (Z039 and Z038).

Compared to the result from our previous study, showing that deaths among 112 patients with non-specific diagnoses constituted 20% of the overall number of deaths after 30 days [3], this study confirmed the high mortality, also on the non-specific sub-diagnostic level both in proportions and in the large number of persons. However, we did not find any good explanations for these deaths apart from ‘suspected cardiovascular disease’ (Z035) and ‘suspected nervous disease’ (Z033).

Thus, the large number of non-specific diagnoses among patients brought to the hospital after calling the national emergency number does not represent only minor illness, and we cannot confirm that they represent unnecessary ambulance transports, even though such cases might be present in this patient group.

Worldwide the need for emergency care is rising and many studies including data from Bavaria, U.K., the U.S., Australia, New Zeeland, and Canada [14–16] confirm increased demand for ambulances.

## Conclusion

We found that sub-diagnosis ‘unspecified disease’ (Z039) constituted nearly half of the non-specific sub-diagnoses among patients brought to the hospital by ambulances after 112 calls. This is in line with several other studies that found similar large proportions of non-specific diagnoses. Furthermore, we studied the outcome and found considerable mortality among patients with non-specific diagnoses, even at the sub-diagnostic level. The results from this population-based study can be generalized to countries with similar prehospital patient populations. However, the diagnostic pattern of prehospital patients is not well known. We suppose generalizability probably is towards the Scandinavian countries, Germany and may be to other European countries.

## Data Availability

As the study include sensitive patient information, restrictions apply to the availability of data that is not publicly available. However, researchers interested in the data can seek approval from the Danish Patient Safety Authority. Having obtained approval, researchers can request data from the Centre for Prehospital and Emergency Research, Aalborg Denmark.

## Abbreviations

Chapter R: Symptoms, signs and abnormal clinical and laboratory findings, not elsewhere classified
Chapter Z: Factors influencing health status and contact with health services
CI: Confidence interval
CPR: Civil personal registration
EMCC: Emergency Medical Coordination Center
ICD-10: The Danish version of the International Statistical Classification of Diseases and related health problems, 10th Edition
PAS: Patient Administration System
